# Accuracy of citrulline, I-FABP and d-lactate in the diagnosis of acute mesenteric ischemia

**DOI:** 10.1038/s41598-021-98012-w

**Published:** 2021-09-23

**Authors:** Alexandre Nuzzo, Kevin Guedj, Sonja Curac, Claude Hercend, Claude Bendavid, Nathalie Gault, Alexy Tran-Dinh, Maxime Ronot, Antonino Nicoletti, Yoram Bouhnik, Yves Castier, Olivier Corcos, Katell Peoc’h, The SURVI (Structure d’URgences Vasculaires Intestinales) Research Group (French Intestinal Stroke Center), Audrey Huguet, Carmen Stefanescu, Xavier Treton, Francisca Joly, Lore Billiauws, Annick Hamon, Aureline Boitet, Céline Lekhal, David Deutsch, Elsa Oiknin, Laura Cohen, Gabriel Marcellier, Jean Senemaud, Felix Corre, Damien Soudan, Cosmin Voican, Jean-Baptiste Leclère, Jules Iquilles, Lucas Raynaud, Luisa Paulatto, Manon Haas, Mathieu Uzzan, Mathilde Cohen, Sara Tadbiri, Servane Prevot, Yves Panis, Alice Frontali, Simon Msika, Lara Ribeiro, Lionel Rebibo, Konstantinos Arapis, Marion Orville, Annie Sibert, Pauline Copin, Magaly Zappa, Marco Dioguardi Burgio, Valérie Vilgrain, Caroline Bertin, Anne Kerbaol, Wassim Allaham, Quentin Pellenc, Arnaud Roussel, Pierre Cerceau, Iannis Ben Abdallah, Antoine Girault, Pierre Mordant, Romain De Blic, Catherine Paugam, Emmanuel Weiss, Paer-Selim Abback, Isabelle Enriquez, Sylvie Janny, Helene Bout, Mikhael Giabicani, Marina Achouf, Bénédicte Grigoresco, Linda Koy Ear, Sonja Curac, Agnès Cachier, Aurelie Plessier, Pierre-Emmanuel Rautou, Dominique Valla, Audrey Payancé, Alain Sauvanet, Safi Dokmak, Federica Dondero, Ailton Sepulveda, Olivier Farges, Beatrice Aussilhou, Bénédicte Jais, Dominique Cazals-Hatem, Emmanuelle De Raucourt, Larbi Boudaoud, Catherine Trichet, Herve Puy, Nathalie Pons-Kerjean, Jeanick Stocco, Julie Bataille, Valérie Bouton, Philippe Montravers, Pascal Augustin, Brice Lortat Jacob, Jean-Baptiste Michel, Dominique Gauguier, Marc-Emmanuel Dumas, François Brial, Antonis Myridakis, Laura Martinez-Gili, Michael Olanipekun, Estelle Marcault, Cindie Nilusmas, Anne Barnier, Aminata Souare

**Affiliations:** 1 APHP, Department of Gastroenterology, IBD and Intestinal Failure, Intestinal Stroke Center, Structure d’Urgences Vasculaires Intestinales (SURVI), Hôpital Beaujon, 100 Bd du Général Leclerc, 92110 Clichy, France; 2 Laboratory for Vascular Translational Science, INSERM UMR 1148, Université de Paris, 75018 Paris, France; 3 APHP, Emergency Department, Beaujon Hospital, 92110 Clichy, France; 4 APHP, Department of Clinical Biochemistry, Beaujon Hospital, 92110 Clichy, France; 5 Institut NuMeCan, INSERM U1241/CHU Rennes/INRA, Université de Rennes, 35000 Rennes, France; 6 APHP, Department of Epidemiology, Biostatistics and Clinical Research, Bichat Hospital, 75018 Paris, France; 7 INSERM CIC-EC 1425, Hôpital Bichat, 75018 Paris, France; 8 APHP, Intensive Care Unit, Bichat Hospital, 75018 Paris, France; 9 APHP, Department of Radiology, Beaujon Hospital, 92110 Clichy, France; 10 Centre de Recherche sur l’Inflammation (CRI), INSERM UMR 1149, Université de Paris, 75018 Paris, France; 11 APHP, Department of Vascular Surgery, Bichat Hospital, 75018 Paris, France; 12 Beaujon-Bichat Hospitals, Paris, France; 13 Assistance Publique-Hôpitaux de Paris, Université de Paris, Paris, France

**Keywords:** Biomarkers, Cardiology, Diseases, Gastroenterology

## Abstract

Early diagnosis of acute mesenteric ischemia (AMI) remains a clinical challenge, and no biomarker has been consistently validated. We aimed to assess the accuracy of three promising circulating biomarkers for diagnosing AMI—citrulline, intestinal fatty acid-binding protein (I-FABP), and d-lactate. A cross-sectional diagnostic study enrolled AMI patients admitted to the intestinal stroke center and controls with acute abdominal pain of another origin. We included 129 patients—50 AMI and 79 controls. Plasma citrulline concentrations were significantly lower in AMI patients compared to the controls [15.3 μmol/L (12.0–26.0) vs. 23.3 μmol/L (18.3–29.8), *p* = 0.001]. However, the area under the receiver operating curves (AUROC) for the diagnosis of AMI by Citrulline was low: 0.68 (95% confidence interval = 0.58–0.78). No statistical difference was found in plasma I-FABP and plasma d-lactate concentrations between the AMI and control groups, with an AUROC of 0.44, and 0.40, respectively. In this large cross-sectional study, citrulline, I-FABP, and d-lactate failed to differentiate patients with AMI from patients with acute abdominal pain of another origin. Further research should focus on the discovery of new biomarkers.

## Introduction

Early management of acute mesenteric ischemia (AMI) can avoid fatal outcomes and prevent related short bowel syndrome^1–4^. To this end, there is a dire need for tools to establish an immediate diagnosis. Mortality and intestinal resection rates have remained unchanged for decades despite the progress made in radiology, endovascular procedures, and intensive care medicine. However, recent reports suggest improved outcomes for patients if diagnosis and standardized multidisciplinary expert care are provided at an early stage^1,5–7^. Indeed, early AMI is a fully reversible condition, as opposed to "advanced" AMI with irreversible transmural necrosis^2,8^. However, AMI patients present unspecific acute abdominal pain, which renders clinical suspicion and identification challenging and can often lead to missed or delayed diagnosis and care^2,9^. Moreover, AMI may be underdiagnosed on contrast-enhanced computed tomography (CT) of the acute abdomen when the suspicion is not evoked^10–12^.

While biological abnormalities—such as leucocytosis or lactic acidosis—have been documented in patients with AMI, their performance to establish the early diagnosis is poor^4,13^. The high complexity of the layered intestinal wall structure increases the diversity of the proteins and metabolites released in AMI. Their hepatic metabolism through the hepatic portal system results in substantial overlap with liver proteins and metabolites. These factors, along with the heterogeneity of the disease, explain why identifying clinically reliable biological early markers of AMI has been unsuccessful so far^2,4,9^.

Three blood biomarkers have gained attention over the past decades: citrulline, a marker of enterocyte function; I-FABP, a marker of enterocyte damage; and d-lactate, a marker of intestinal barrier dysfunction and microbial translocation^4,14–16^. As a result, these tests are increasingly used in basic and clinical research as indirect markers of an ischemic intestinal injury in a broad range of emergency clinical settings. However, to date, their alleged diagnostic performances have only been assessed in small heterogeneous cohorts. Besides, conflicting results have been reported, and most of the studies consist of preoperative data in late-stage necrotic AMI patients^3^. As a consequence, the accuracy of these three biomarkers in identifying early-stage AMI remains to be conclusively tested^17,18^.

The aim of this cross-sectional diagnostic study was to assess the accuracy of these three promising circulating biomarker candidates—citrulline, I-FABP, and d-lactate—for the diagnosis of AMI.

## Methods

### Study design and setting

Following the results of a pilot study showing an improvement in survival and lower resection rates^1^, we created an intestinal stroke center (ISC) that provides 24/7 standardized multimodal and multidisciplinary care to AMI patients referred from the Paris region^19^. Since the creation of this center, we prospectively enrolled patients from the ISC department and the emergency room (ER) department who undergo a contrast-enhanced CT-scan for acute abdominal pain as part of the SURVIBIO cross-sectional diagnostic study. The patients' blood samples were collected on admission and stored in a biobank for further biomarker analysis and research. We used the blood samples from the biobank to assess the performance of three circulating candidate biomarkers—citrulline, I-FABP, and d-lactate—in the diagnosis of AMI. The study was approved by the Ethics Committee of Paris-Nord Val de Seine University Hospitals. The study complies with the Standards for Reporting Diagnostic Accuracy (STARD) guidelines^20^. Written informed consent was obtained from all patients.

### Patients and controls

**Figure 1:**
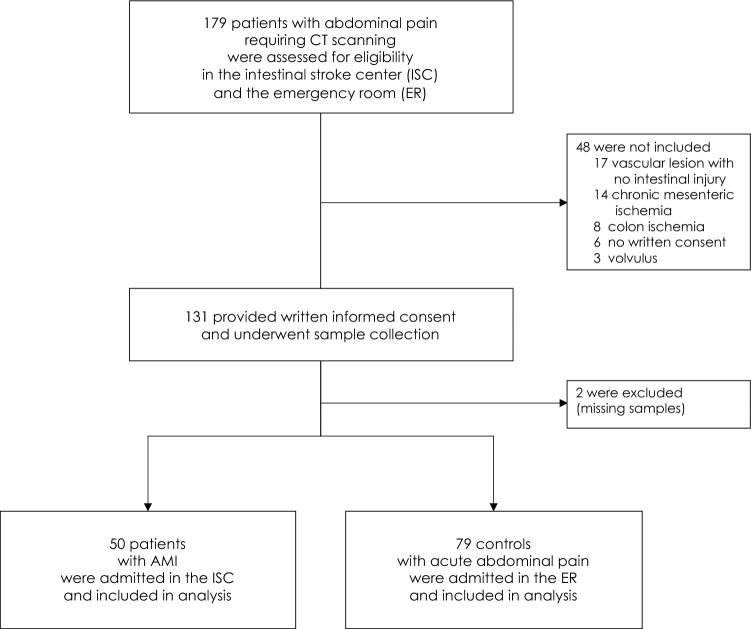
Flowchart of AMI patients and controls: screening and selection. Abbreviations: *AMI* acute mesenteric ischemia, *CT* contrast-enhanced computed tomography.

From January 4, 2016, to March 5, 2018, prospective patients who presented with acute abdominal pain requiring a contrast-enhanced CT scan to the ER department or referred to the ISC department were evaluated for inclusion in the SURVIBIO diagnostic study. Patients with AMI were admitted to the ISC, whereas those in whom the diagnosis of AMI was ruled out (controls) were admitted to the emergency room (see patient flowchart, Fig. 1). Patients presenting with a diagnosis of left-sided colon ischemia without small bowel injury, chronic mesenteric ischemia without acute injury, vascular lesions with no small bowel injury, or strangulated bowel obstruction were not included (see patient flowchart, Fig. 1).

AMI was defined by the association of (1) acute clinical, biological, and/or contrast-enhanced CT features of bowel injury, (2) vascular insufficiency (occlusive or non-occlusive) of the celiac trunk and/or the superior mesentery artery and/or superior mesenteric vein, and (3) the absence of an alternative diagnosis^6^. The diagnosis was subsequently confirmed by histology following intestinal resection^8^.

The diagnosis of AMI was ruled in or out by the CT scan, and alternative final diagnoses were based on clinical, biological, and CT findings. All the patients' clinical records, CT-scans, and pathologic specimens were reviewed in a multidisciplinary meeting, including gastroenterologists, radiologists, digestive and vascular surgeons, and intensivists, all experts in digestive vascular diseases, to avoid diagnostic misclassification before any blood sample analysis.

All the AMI patients were managed by a standardized multimodal and multidisciplinary approach in our intestinal stroke center, as previously described^1^. Briefly, the patients were systematically administered oral antibiotics and antithrombotics^1,6^, and emergency endovascular revascularization of arterial AMI was performed whenever technically feasible. Alternatively, open surgical revascularization was performed. Bowel viability was evaluated by laparotomy, decided based on published risk factors for irreversible transmural intestinal necrosis (occurrence of organ failure, elevated plasma lactate concentrations, small bowel dilatation, or perforation on CT)^8^. Irreversible transmural intestinal necrosis was confirmed upon pathological assessment.

### AMI subgroups

AMI patients were characterized depending on the cause of the splanchnic vascular insufficiency as being arterial AMI (including arterial stenosis, thrombosis or embolus) or venous AMI (due to portal and/or mesenteric vein thrombosis or compression). Patients presenting with or without irreversible transmural intestinal necrosis were included in the late necrotic or early AMI subgroups, respectively. The presence of irreversible intestinal ischemic injury was defined, as previously proposed^8^, by: (a) pathology assessment as anoxic, extensive, transmural necrosis with hemorrhagic and/or gangrenous infarction or (b) evidence of bowel perforation on CT evaluation or (c) extensive necrosis assessed during open-close laparotomy procedures in unresected patients. Patients with superficial non-transmural ischemic necrosis upon pathological assessment and those who recovered from AMI with no need for digestive surgery were considered not to have irreversible transmural intestinal necrosis^8^.

### Data collection and processing

Routine baseline clinical and biological characteristics were collected upon admission for all patients: age, gender, body mass index (BMI), history of cardiovascular disease or risk factors, general and digestive clinical signs, and common biological features. The origin of AMI (arterial—thrombotic or embolic—venous, or non-occlusive) was specified based on the patient's records, CT-scan, and pathologic review.

Blood samples were collected from all the patients on admission and stored for further biomarker analysis. Blood samples were collected in appropriate vacutainers before immediate centrifugation at 1500 × g for 15 min at room temperature, and isolated plasma samples underwent subsequent storage at − 80 °C until further analysis.

Blood samples were collected in appropriate vacutainers before immediate centrifugation at room temperature, and isolated plasma samples have undergone subsequent storage.

Biomarker measurements were performed in 2019 after the final diagnosis classification had been made in all patients. After randomization of all samples, Citrulline, d-lactate, and I-FABP levels were measured on plasma collected in heparin, oxalate, and EDTA-treated tubes, respectively.

I-FABP concentrations were measured in plasma using two different ELISA kits [Hycult Bioteck, Uden, The Netherlands, HK40602, range: 0–3000 ng/L^21,22^; and R&D Systems, Liey, DY3078, linearity range: 0–1000 ng/L^23^] according to the manufacturer's instructions. We used two quality controls (sample pool)—at the beginning and the end of the plates. Briefly, samples and standards were incubated in 96-well microtiter plates coated with antibodies recognizing human I-FABP. The biotinylated secondary antibody was then added to the wells. After washes, streptavidin-peroxidase conjugate, which binds the biotinylated secondary antibody, was added and reacted with tetramethylbenzidine (TMB) substrate. The absorbance was then measured with a spectrophotometer at 450 nm (Infinite 200 PEO, TECAN).

Citrulline plasma levels were assayed using an ultra-performance liquid chromatography-mass spectrometer (UPLC-MS; Xevo TQS, Waters). We first conducted a two-step extraction: 100 μL of heparin plasma were added to 300 μL of acetonitrile (ACN; VWR) with 0.05% citrulline C13 (Cambridge Isotope laboratories Inc.)and 1% formic acid (FA; VWR) to precipitate proteins. After 5 min of mechanical agitation, the mixes were centrifuged at 13,000 × g for 5 min at room temperature. Then, 300 µL of the supernatant was loaded onto an Ostro Plate (Waters) and extracted under positive pressure. The eluate was diluted 1:2 in ACN/H20 (90:10), and 2 µL were injected in the UPLC-MS. The mobile phase A was water with ammonium formate 10 mM (A.F.; VWR) and 0.15% FA, and the mobile phase B was A.F. 10 mM in ACN with 0.15% FA. The retention time was 1.43 min on the Xevo TQS, Waters. The results are presented as an average of two measurements (duplicate). If a coefficient of variation higher than 10% was observed between two measures, the sample was reanalyzed.

d-lactate plasma concentration was assayed by a kinetic spectrophotometric method using an adapted method of the Biosentec d-Lactic acid kit on a Cobas C111 analyzer (Roche). Briefly, the reaction used an endpoint analysis of the following reaction, catalyzed by the d-lactate dehydrogenase: Lactate + NAD → Pyruvate + NADH + H + Pyruvate + Glutamate → Alanine + α-ketoglutarate. NADH was measured at 340 nM. Low-and high-level quality controls were included at the beginning and the end of each batch.

### Statistical analysis

For each of the continuous variables, we report the median and the interquartile range (IQR). Categorical variables are expressed as the number of observations and percentages. Normally distributed quantitative data were analyzed with the Student t-test. Mann–Whitney U test was used otherwise. Subgroup analyses were performed with the use of the Kruskal–Wallis tests for skewed distributions. When the result of a global test was significant (*p* < 0.05), post hoc Bonferroni-corrected pairwise comparisons were performed. Qualitative data were compared with either the Pearson χ^2^ test or the Fisher exact test, depending on the sample size. We determined that the enrollment of 50 patients in each group would provide a power of more than 95% for assessing diagnostic tests with AUC ≥ 0.70^24^. The diagnostic accuracy of each biomarker was evaluated by analyzing the receiver operating curve (ROC) with the calculation of the area under the ROC (AUROC). The maximum value of Youden's index was used as a criterion for selecting the optimal cut-off value of each biomarker, reported with its associated sensitivity, specificity, negative predictive values (NPV), and positive predictive values (PPV). All tests were two-sided. No imputation of missing data was performed. Analyses were performed using the Statistical Package for the Social Sciences (SPSS) for Mac OSX software (version 23.0, Chicago, IL, USA) and the pROC package^25^ in R software, version 3.6.2 (R Foundation for Statistical Computing).

## Results

### Characteristics of study subjects

A total of 179 patients with acute abdominal pain requiring a contrast-enhanced CT-scan were assessed for eligibility (Fig. 1). We included and collected blood samples from 131 patients, including 50 admitted to our intestinal stroke center for AMI and 79 admitted to the emergency room for non-AMI acute abdominal pain (see Flowchart, Fig. 1). We excluded 2 patients (controls) with missing samples upon biological analysis. The baseline characteristics of both populations are summarized in Table 1. The final diagnosis of the controls is presented in Table 2. Patients with AMI [median age: 65 years (IQR 55–75), 38% of women] included arterial and venous causes in 66% and 34% of cases, respectively. None of the patients included had non-occlusive AMI. AMI occurred in seven patients with past history of chronic mesenteric ischemia. Patients with AMI were significantly older, had a higher BMI, and were more likely to have risk factors or a history of cardiovascular disease than controls (Table 1). AMI patients were also more likely to present hematochezia, guarding, and organ dysfunction (as measured by a total Sequential Organ-Failure Assessment (SOFA) score ≥ 2)^26^ and a higher white blood cell count at baseline. Other clinical and biological characteristics, including l-lactate levels, did not differ significantly (Table 1). AMI patients were referred from primary care hospitals within a median of 8 h (range 6–12). After admission in the intestinal stroke center, AMI patients received antiplatelet therapy (n = 33, 100% arterial AMI), anticoagulants (n = 50, 100%), oral antibiotics (n = 49, 98%), and intravenous antibiotics (n = 21, 42%). Emergency revascularization was performed in 29 patients (88% of arterial AMI patients).

**Table 1:** Baseline characteristics of AMI patients and controls.

	AMI patients n = 50 (%)	Controls n = 79 (%)	*p* value
Age, years^a^	65 (55–75)	45 (35–71)	< 0.001
BMI, kg/m^2a^	27 (21–33)	22 (20–24)	< 0.001
Female	19 (38)	31 (39)	0.89
**Atherosclerosis risk factors**			
Tobacco use	23 (46)	16 (20)	0.002
Arterial hypertension	28 (56)	20 (25)	< 0.001
Dyslipidemia	19 (38)	12 (15)	0.003
Diabetes mellitus	12 (24)	5 (6)	0.004
**Cardiovascular history**			
Myocardial ischemia	9 (18)	5 (6)	0.04
Stroke	6 (12)	5 (6)	0.34
Limb ischemia	9 (18)	2 (3)	0.003
Atrial fibrillation	11 (22)	3 (4)	0.001
Deep vein thrombosis	3 (6)	4 (5)	1.00
Pulmonary embolism	5 (10)	4 (5)	0.31
**Other comorbidities**			
Chronic kidney disease	1 (2)	2 (3)	1.00
Cirrhosis	4 (8)	4 (5)	0.71
Abdominal surgery	27 (54)	37 (47)	0.43
**Clinical features**			
Temperature^a^	37.0 (36.3–37.1)	36.8 (36.5–37.5)	0.49
Mean arterial pressure^a^	100.3 (90.8–110.3)	96.0 (83.7–105.7)	0.17
Vomiting	21 (42)	39 (49)	0.41
Diarrhea	12 (24)	13 (17)	0.29
Hematochezia	8 (16)	3 (4)	0.02
Guarding	16 (32)	13 (17)	0.04
Organ dysfunction (total SOFA score ≥ 2)	15 (30)	8 (10)	0.004
**Biological features**^a^			
White blood cell count, 10^9^/L	12.3 (9.3–18.3)	10.6 (8.0–14.0)	0.02
Platelet count, 10^9^/L	268 (177–367)	269 (231–333)	0.74
Hemoglobin, g/dL	12.3 (11.5–15.1)	13.6 (12.4–14.8)	0.14
l-Lactate levels, mmol/L	1.49 (1.02–2.41)	1.37 (0.83–2.44)	0.56
Creatinine, µmol/L	70 (62–100)	70 (62–84)	0.86
AST, IU/L	26 (19–38)	27 (21–41)	0.29

Abbreviations: *AMI* acute mesenteric ischemia, *BMI* body mass index, *SOFA* sequential organ failure assessment, *AST* aspartate aminotransferase, *IU* international units.

^a^Median (inter quartile range).

**Table 2:** Etiological diagnosis of the control group with acute abdominal pain.

Diagnosis	n = 79 (%)
**Infectious**	18 (23)
Diverticulitis	9
Appendicitis	4
Other causes of peritonitis/abdominal abscess	5
**Inflammatory**	15 (19)
Intra-abdominal neoplasm progression	8
IBD flares	7
Bowel obstruction (non-strangulated)	13 (16)
Functional GI disorders (reflux, diarrhea, constipation, etc.)	12 (15)
**Biliopancreatic tract**	10 (13)
Pancreatitis	7
Biliary complications	3
Urogenital	10 (13)
Invasive meningococcal disease	1 (1)

Abbreviations: *IBD* inflammatory bowel disease, *GI* gastro-intestinal.

### Main results

**Figure 2:**
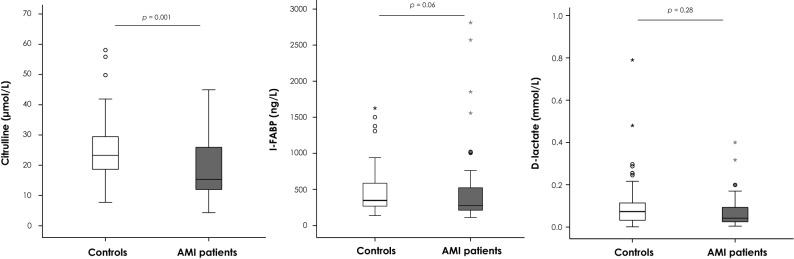
Citrulline, I-FABP and d-lactate plasma concentrations in AMI patients and controls. Abbreviations: *I-FABP* intestinal fatty-acid binding protein, *AMI* acute mesenteric ischemia. Comparison of acute abdominal pain controls (n = 79) and patients with AMI (n = 50). The horizontal line in the boxes represents the median, and the bottom and top of the boxes the 25th and 75th percentiles, respectively. I bars represent the upper adjacent value (75th percentile plus 1.5 times the interquartile range) and the lower adjacent value (corresponding formula below the 25th percentile). Outliers are represented by dots (outside of the I bars) and extreme outliers by asterisks (outside of the 75th percentile plus 3 times the interquartile range, or the 25th percentile minus 3 times the interquartile range).

Median plasma citrulline concentrations were significantly lower in the AMI patients compared to the controls [15.3 μmol/L (IQR 12.0–26.0) vs. 23.3 μmol/L (18.3–29.8), *p* = 0.001], although the two distributions broadly overlapped (Fig. 2). Plasma I-FABP concentrations tended to be lower in the AMI population compared to the controls using Hycult kit [278 ng/L (209–544) vs. 348 ng/L (268–587), *p* = 0.06]. We then confirmed a similar trend using the R&D kit (Supplementary Data Fig. 1). Results obtained with the Hycult kit were further used for statistical analyses.

Plasma d-lactate concentrations did not significantly differ between AMI patients and controls [0.042 mmol/L (0.025–0.095) vs. 0.073 mmol/L (0.031–0.115), *p* = 0.28)]. Performances of the three biomarkers for the diagnosis of AMI are summarized in Table 3.

**Table 3:** Diagnostic performance of plasma citrulline, I-FABP, and d-lactate for the diagnosis of acute mesenteric ischemia.

Biomarker	AUROC (95%CI)	Cut-off value	Sensitivity	Specificity	PPV	NPV	Youden index
Citrulline (µmol/L)	0.679 (0.577–0.781)	16.6	0.56	0.84	0.68	0.75	0.40
I-FABP (ng/L)	0.401 (0.291–0.512)	974	0.15	0.95	0.64	0.64	0.10
d-Lactate (mmol/L)	0.438 (0.328–0.547)	0.012	0.98	0.17	0.43	0.92	0.14

PPV and NPV were calculated based on a prevalence of AMI = 0.39 in the study population.

Abbreviations: *AUROC* area under the receiver operating curve, *PPV* positive predictive value, *NPV* negative predictive value, *CI* confidence interval, *I-FABP* intestinal fatty acid-binding protein.

### Arterial versus venous AMI subgroups

Thirty-three patients (66%) presented with arterial AMI, and 17 patients (34%) presented with venous AMI. Compared to the controls, median plasma citrulline was significantly lower in patients with arterial AMI [14.5 μmol/L (11.1–29.1) vs. 23.3 μmol/L (18.3–29.8), *p* = 0.01] and slightly lower in patients with venous AMI [16.6 μmol/L (13.0–21.2) vs. 23.3 μmol/L (18.3–29.8), *p* = 0.06]. We did not observe significant differences in the concentration of plasma I-FABP in arterial and venous AMI versus control patients [281 ng/L (169–647) and 262 ng/L (248–487) vs. 348 ng/L (268–587), respectively, Kruskal–Wallis test *p* = 0.21]. Neither did we observe significant differences in the concentration of plasma d-Lactate concentrations [0.054 mmol/L (0.033–0.107) in arterial AMI and 0.025 mmol/L (0.022–0.088) in venous AMI vs. 0.073 mmol/L (0.031–0.115) in controls, Kruskal–Wallis test *p* = 0.24] (Supplemental data Fig. 2).

### Early versus late necrotic AMI subgroups

During the follow-up period, 17 of the AMI patients (34%) required a laparotomy, and 12 (24%) required bowel resection. Irreversible transmural intestinal necrosis was pathologically confirmed in 10 of the 12 resected cases. Four additional patients had undergone open-close procedures (i.e., without performing bowel resection) showing visible extensive dark necrosis and were included in the late necrotic AMI subgroup (n = 14). Patients with superficial and non-transmural necrosis (n = 2), as well as those who recovered from AMI with no need for bowel resection (n = 34), were included in the early AMI subgroup (n = 36).

**Figure 3:**
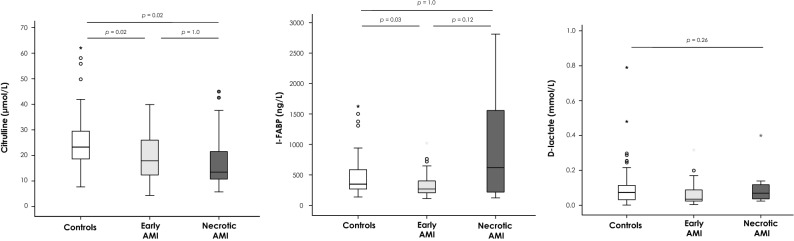
Citrulline, I-FABP, and d-lactate blood concentrations according to the severity of acute mesenteric ischemia (early or late—necrotic—ischemia). Abbreviations: *AMI* acute mesenteric ischemia, *I-FABP* intestinal fatty-acid binding protein. Comparison of acute abdominal pain controls (n = 79) and patients with early AMI (n = 36), and necrotic AMI (n = 14). Analyses were performed with the use of the Kruskal–Wallis test. When the result of a global test was significant (*p* < 0.05), post hoc Bonferroni-corrected pairwise comparisons were performed. The horizontal line in the boxes represents the median, and the bottom and top of the boxes, the 25th and 75th percentiles, respectively. I bars represent the upper adjacent value (75th percentile plus 1.5 times the interquartile range) and the lower adjacent value (corresponding formula below the 25th percentile). Outliers are represented by dots (outside of the I bars) and extreme outliers by asterisks (outside of the 75th percentile plus 3 times the interquartile range, or the 25th percentile minus 3 times the interquartile range).

As presented in Fig. 3, plasma citrulline concentrations were significantly lower in both late necrotic AMI [13.5 (10.3–25.5) µmol/L, *p* = 0.02] and early AMI patients [17.9 (12.3–26.0) µmol/L, *p* = 0.02] as compared to controls [23.3 (18.3–29.8) μmol/L]. However, the comparison failed to discriminate late necrotic from early AMI patients. We observed a trend of higher plasma I-FABP concentrations in late necrotic AMI patients [619 ng/L (212–1632)] compared to early AMI patients [268 ng/L (205–403), *p* = 0.12] and controls [348 ng/L (268–587), *p* = 1.0]. However, I-FABP levels were significantly lower in early AMI patients compared to controls (*p* = 0.03). We did not find any significant difference in plasma d-lactate concentrations between late necrotic AMI patients [0.069 mmol/L (0.036–0.128)] or either early AMI patients or controls [0.035 mmol/L (0.024–0.091) and 0.073 mmol/L (0.031–0.115), respectively, Kruskal–Wallis test *p* = 0.26].

## Discussion

This cross-sectional diagnostic study of 129 patients admitted for acute abdominal pain found that the three most promising circulating biomarker candidates for AMI—citrulline, I-FABP, and d-lactate—were neither sensitive nor specific enough for differential diagnosis of AMI: I-FABP and d-lactate displayed low AUROC values, and citrulline assays had low sensitivity. These results contrast with published reports^4,14–16^. This could be explained by two critical differences in the experimental designs. Firstly, previous studies enrolled severe AMI patients at a late necrotic surgical stage. This may have created a selection bias leading to an overestimated performance of the studied biomarkers, not generalizable to earlier stages of the disease. Secondly, in most of the studies published so far, only a restricted number of AMI patients were included. They were merged with heterogeneous pathologic conditions labeled “ischemic bowel diseases”, such as strangulated bowel obstructions or left-side colon ischemia with no small bowel injury. This may have led to an overestimation of the specificity of the studied biomarkers for the diagnosis of genuine vascular-related AMI. For instance, Shi et al. studied 39 patients with “acute intestinal ischemic disease,” including 26 with strangulated bowel obstruction, six with colon ischemia with no small bowel injury, and only seven patients with genuine AMI at the infarction stage^27^. Similarly, Kanda et al. studied 52 preoperative patients with “small bowel ischemia,” including 45 cases of strangulated bowel or incarcerated hernia and only three patients with late necrotic AMI^28^. In the present study, we included a large and homogeneous population of 50 patients with confirmed arterial and venous AMI, treated with a standardized care protocol in an intestinal stroke center, enrolled on admission at the time of diagnosis, and at an early “non-transmural” AMI stage in 72% of cases. Therefore, our design yields reliable results on clinically applicable AMI biomarkers in its emergency “real-life” early-stage diagnosis.

I-FABP is a cytosolic protein expressed by mature enterocytes at the tip of the small bowel villi, which is the first region affected by ischemia^29^. In the past decades, a few studies on human samples have identified I-FABP as the most promising biomarker for diagnosing AMI. The recent meta-analysis by Sun et al. calculated a pooled sensitivity, specificity, and AUROC of 0.80, 0.85, and 0.86, respectively^15^. Nevertheless, it should be noted that only 60 of the 1246 patients studied in this meta-analysis had genuine vascular-related AMI, mainly at the infarction stage (after a careful review of seven of the nine English-language studies included)^15^. Furthermore, this meta-analysis included studies using various methods for I-FABP measurements and various blood sample types that could be significant confounding factors.

Although we observed higher I-FABP plasma concentrations in late necrotic AMI patients, its discriminative diagnostic performance for AMI was not confirmed. This result was supported by another ELISA assay to exclude any analytical bias (see Supplemental Data Fig. 1). We even observed higher I-FABP concentrations in controls in comparison with early-stage AMI patients. This observation is also in line with numerous published studies that observed high I-FABP blood concentrations in various other non-ischemic gastro-intestinal conditions such as Crohn's disease^30^, celiac disease^31,32^, acute pancreatitis^33,34^, or abdominal surgery/infection^35^.

d-Lactate is the stereoisomer of l-lactate and is almost exclusively produced by microbial fermentation in the gastro-intestinal tract. Consequently, animal experiments and a few clinical studies identified this molecule as a marker of intestinal barrier dysfunction and microbial translocation or overgrowth^36,37^. However, its accuracy has been questioned by studies such as the ones by Van Der Voort et al.^38^ and Block et al.^39^, reporting low (23%) specificity, consistent with the present findings.

Citrulline is an amino acid synthesized from glutamine by small bowel enterocytes. It is considered as a functional biomarker of total enterocyte mass and intestinal failure. Low citrulline plasma levels have been found to correlate with remnant small bowel length and intestinal failure in patients with short bowel syndrome^40^ and with the severity of villous atrophy in patients with celiac disease^41^. In a prospective single-center observational study that included adults without small bowel disease and chronic renal failure consecutively admitted in an intensive care unit, citrulline plasma concentrations were lower for the 24 h following the circulatory shock^42^. Concentrations < 10 µmol/L were highlighted as an independent risk factor of mortality, suggesting that low plasma citrulline at 24 h could be a marker of acute intestinal failure in critically ill patients. We observed significantly lower citrulline plasma levels in patients with AMI compared to the controls, consistent with prior reports^16,43^. As already reported—but here in a larger cohort—we confirm that the sensitivity of citrulline is insufficient in this setting. Our findings are consistent with the results obtained by Kulu et al*.* They found a sensitivity of 39% for citrulline for the identification of AMI in 23 patients, with the best cut-off value at 15.8 µmol/L^43^. Altogether, this corroborates that citrulline displays a low diagnostic performance for AMI, which, regrettably, limits its usefulness: sensitivity is more important than specificity for AMI given the severe consequences of misdiagnosis and offered that confirmation by a contrast-enhanced CT scan remains mandatory to guide emergency treatment (type of revascularization, need for intestinal resection).

A few limitations derived from the design of the present study deserve to be mentioned. First, we chose not to include patients with strangulated bowel obstruction, left-sided colon ischemia, and mesenteric vessel occlusion without evidence of acute minor bowel injury from both patients and control groups. Although these conditions may share pathophysiological processes comparable with AMI, they are different diseases with different prognoses. Although this might decrease the generalizability of our findings to these other conditions, we believe this selection is critical to reduce variability and avoid analytical bias, which are the main shortcomings of available reports in this field of research^3^. Nonetheless, our results are based on the largest population of well-characterized and homogeneous AMI patients. This is crucial as the diagnosis of AMI amongst emergency patients admitted for acute abdominal pain remains a critical clinical challenge^3^, and our findings indicate that the three tested biomarkers do not reach a sufficient diagnostic performance level. Moreover, as compared with previously published studies, we used two different assays for I-FABP, as well as two robust, standardized methods for both citrulline and d-lactate measurements. Furthermore, as no patients with non-occlusive mesenteric ischemia (NOMI) were included in the present study, our results are not generalizable to this specific cause of mesenteric ischemia. Indeed, NOMI is usually the complication of an underlying severe disease causing systemic hypoperfusion in intensive care patients, and such patients were not the target population of this diagnostic study.

In conclusion, Citrulline, I-FABP, and d-lactate failed to identify AMI from acute abdominal pain controls in a large cross-sectional diagnostic study from an Intestinal Stroke Center. Early diagnosis remains a critical clinical and research challenge as it would allow early management and consequently improve the dire prognosis of AMI. We believe that relevant new biomarkers may be identified using non-targeted multi-omics discovery approaches in large cohorts of early-stage AMI patients admitted in intestinal stroke centers to meet this challenge. This objective is of the utmost importance since the introduction of new biomarkers may genuinely alter the epidemiology, diagnosis, management, and outcome of AMI.

## Supplementary Information

Supplementary Figures.

## Data Availability

Research data are not shared.
