# Supplementary material for: Accuracy of citrulline, I-FABP and d-lactate in the diagnosis of acute mesenteric ischemia
Source: Sci Rep. 2021 Sep 23;11:18929. doi: 10.1038/s41598-021-98012-w (PMC8460675; doi:10.1038/s41598-021-98012-w)
Supplement: Supplementary file 1 — Supplementary Figures. [file 41598_2021_98012_MOESM1_ESM.docx]

**Supplemental Figure 1. I-FABP plasma concentrations in AMI patients and controls using the R&D Systems ELISA kit**

I-FABP concentrations were measured in plasma using the ELISA Kits from R&D Systems, Liey, DY3078, (linearity range: 0 – 1000 ng/L).

No significant difference was found in plasma I-FABP concentrations between AMI patients compared to the controls [220 ng/L (152-445) vs 258 ng/L (178-420), p=0.30].

The area under the receiver operating curve (AUROC) for the diagnosis of AMI by plasma I-FABP was 0.44 (95%CI = 0.33 – 0.56)

**Supplemental Figure 2. Citrulline, I-FABP, and D-lactate plasma concentrations according to the origin of acute mesenteric ischemia (venous or arterial)**

Abbreviations: AMI: acute mesenteric ischemia; I-FABP: intestinal fatty-acid binding protein;

Comparison of acute abdominal pain controls (n=79) and patients with venous AMI (n=17), and arterial AMI (n=33). Analyses were performed with the use of the Kruskal-Wallis test. When the result of a global test was significant (p<0.05), post hoc Bonferroni-corrected pairwise comparisons were performed. The horizontal line in the boxes represents the median, and the bottom and top of the boxes, the 25^th^ and 75^th^ percentiles, respectively. I bars represent the upper adjacent value (75^th^ percentile plus 1.5 times the interquartile range) and the lower adjacent value (corresponding formula below the 25^th^ percentile). Outliers are represented by dots (outside of the I bars) and extreme outliers by asterisks (outside of the 75^th^ percentile plus 3 times the interquartile range, or the 25^th^ percentile minus 3 times the interquartile range).
